# Acknowledgement to Reviewers of *Foods* in 2019

**DOI:** 10.3390/foods9010107

**Published:** 2020-01-19

**Authors:** Foods Editorial Office

**Affiliations:** MDPI, St. Alban-Anlage 66, 4052 Basel, Switzerland

The editorial team greatly appreciates the reviewers who have dedicated their considerable time and expertise to the journal’s rigorous editorial process over the past 12 months, regardless of whether the papers are finally published or not. In 2019, a total of 706 papers were published in the journal, with a median time to first decision of 15 days and a median time from submission to publication of 35 days. The editors would like to express their sincere gratitude to the following reviewers for their generous contribution in 2019:
Abdulkerim, ErogluHaliński, Łukasz P.Parrotta, LuigiAbraham, Elizheeba ChristieHallmann, EwelinaParveen, IffatAbu-Reidah, Ibrahim M.Hamid, NazimahPashikanti, SrinathAcquistucci, RitaHamill, RuthPasławska, MartaAdamczak, MarekHamlin, RobertPasqualone, AntonellaAdamkova, AnnaHammann, SimonPassoth, VolkmarAdedeji, AkinbodeHan, NamSooPatange, ApurvaAdeleye, BerniceHanania, MichelPatarata, LuísAdhikari, Khem BahadurHanga, Mariana Petronela (Petra)Pateiro, MirianAdhikari, KoushikHano, ChristophePatel, AlokAdiletta, GiuseppinaHansen, Egon BechPatel, AnantAdjonu, RandyHanssen, Ole JørgenPatel, AshokÅdnøy, TormodHarasym, JoannaPatelski, PiotrAfonso, Clélia N.Harbourne, NiamhPaterson, R. Russell M.Ageitos, Jose ManuelHardacre, AllanPatila, MichaelaAgić, DejanHarmon, AlisonPaulsen, PeterAgnieszka, SzopaHartman, MariuszPauselli, MarianoAgriopoulou, SofiaHauge, Sigrun J.Pawlak-Lemańska, KatarzynaAgulheiro Santos, Ana CristinaHavlik, JaroslavPazourek, JiriAgyei, DominicHebishy, EssamPeana, Massimiliano F.Ahn, Mok-RyeonHeinen, MirjamPedersen, HenrikAhrens, Richard A.Heldman, DennisPeira, GiovanniAiello, GildaHenchion, MaevePeiretti, Pier GiorgioAires, AlfredoHenen, Morkos A.Pelle, Flavio DellaAkter, Yeasmin AraHenschke, PaulPellegrino, RobertAlamprese, CristinaHermes, Gerben D. A.Peng, MeiAlashi, Adeola M.Hernandez-Hernandez, OswaldoPeng, MengfeiAl-Asmar, AsmaaHerppich, WernerPennone, VincenzoAlba-Elías, FernandoHerrero, BaudilioPenson, PeterAlbergamo, AmbroginaHerrero, HernándezPepino, M. YaninaAlberto Lopes, Joao FilipeHess, JuliePerazzo, AntonioAlencikiene, GitanaHibi, TakaoPereira, CarlaAl-Fatimi, MohamedHill, CameronPereira, Carlos DiasAlfieri, AndreinaHilliou, LoicPereira, J. A.Almajano, María PilarHingley, MartinPereira, Maria De LourdesAlmeida, JoseHocquette, Jean-FrançoisPereira, Olívia R.Almeida, Maria JuditeHoel, SunnivaPerestrelo, RosaAlonso-Calleja, CarlosHoffmann, Klaus H.Perez Nevado, FranciscoAlshannaq, AhmadHolck, Askild LorentzPérez-Burillo, SergioAltemimi, AmmarHollung, KristinPerpetuini, GiorgiaAlvarez Fernandez, María AntoniaHolman, BenjaminPerucka, IrenaAlvarez-Suarez, JoseHomma, TakujiroPeterson, JuliaAmato, MarioHong, JaeheePetrikaite, VilmaAmbrosone, AlfredoHopfer, HelenePetrikova, IvicaAmigo, LourdesHorax, RonnyPetroni, KatiaAmores, AntonioHowell, KatePetropoulos, SpyridonAncuceanu, Robert ViorelHsieh, Chang-WeiPeyrot Des Gachons, CatherineAndersen, Barbara VadHsu, Chia-LinPezzolesi, LauraAndrade, IsabelHsu, Yuan-ManPiątkowska, EwaAndrade, José CarlosHu, ChenlinPiechowiak, TomaszAngelino, DonatoHuang, Chun-YungPielech-Przybylska, KatarzynaAnjos, OféliaHulánková, RadkaPieszka, MarekAnnor, GeorgeIammarino, MarcoPignitter, MarcAnnunziata, LoredanaIbarra-Castanedo, ClementePilkington, Suzanne M.Antolak, HubertIbrahim, HishamPilolli, RosaAntonelli, AndreaIglesias Carres, LisardPilot, RobertoAntonio, Amilcar L.Igne, BenoîtPinheiro, JoaquinaAppell, MichaelIgual, MartaPinho, EvaAraujo, Maria E. M.Iliada, LappaPino, AlessandraArce, LourdesInam Afzal, MuhammadPintado, TatianaAreal Borrego, FranciscoIrakli, MariaPinto, LorisArgyropoulos, DimitriosIseppi, RamonaPiscopo, AmaliaArroyo-López, Francisco NoéIslam, Mohammad ZahirulPiwowarek, KamilArtiaga, LuisItterbeeck, Joost VanPlazzotta, StellaArtieda, Diana AnsorenaIwaniak, AnnaPłotka-Wasylka, JustynaAshrafi, AmirmansoorIweala, OnyinyePoczta-Wajda, AgnieszkaAsioli, DanieleJablonska, EwaPogorzelska-Nowicka, EwelinaAstruc, ThierryJahnke, BenediktPolewski, KrzysztofAtanasov, AtanasJaime, LauraPopovich, DeidreAugustyniak, AdrianJakobusic Brala, CvijetaPossas, AríciaAuh, Joong-HyuckJakubczyk, AnnaPotes, Maria EduardaAung, Wai PhyoJamróz, EwelinaPoucke, Christof VanAvallone, SylvieJamwal, RohitashPouliot, YvesAvi, ShpigelmanJaniszewska-Turak, EmiliaPozo, Elena PeñasAyad, AmiraJanowicz, MonikaPradal, D.Azzini, ElenaJanssens, KimPrado-Cabrero, AlfonsoBahrim, GabrielaJappe, UtaPrester, LjerkaBai, JinheJärvenpää, Eila P.Preti, RaffaellaBaiano, AntoniettaJasicka-Misiak, IzabelaPrgomet, IvaBailly, Jean-DenisJean-Gilles Beaubrun, JuniaPrinz, PhilipBajpai, VivekJedziniak, PiotrPriyadarshini, AnushreeBalawejder, MaciejJefferies, L.K.Proestos, CharalamposBallester, JordiJeromel, AnaProkopov, TsvetkoBalusamy, Sri RenukadeviJeżewska-Zychowicz, MarzenaPrzekora, AgataBalzan, StefaniaJi, ShaofeiPtaszek, AnnaBamba, Bio Sigui BrunoJiang, WaynePulina, GiuseppeBanchero, MauroJiménez, AlbertoPyatkovskyy, TarasBanoub, Joe HJiménez, Edith CoronaPycia, KarolinaBanovic, MarijaJoão Ramalhosa, MariaPyka, AlinaBaranowska, HannaJokić, StelaQiu, BinBarbosa-Pereira, LetriciaJones, Owen GriffithQuaresma, Mário A. G.Barisan, LuiginoJoo, Seon-TeaQuiles, JoanBarrasso, RobertaJordão, António ManuelQuintas, CeliaBarreca, DavideJoyce, AmandaQuirino, Joselito P.Barreiro, Sonia LosadaJózsa, LászlóR. El-Seedi, HeshamBarrett, AnnJukić, MarkoRaak, NorbertBarroca, Maria JoãoJun, Joon-YoungRagupathy, SubramanyamBarros, AntonioJung, SamooelRagusa, AndreaBarth, MargaretJungnickel, HaraldRaheem, BamideleBartoszek, AgnieszkaJusticia, JoséRahmani, DjamelBasiak, EwelinaJuszczak, LesławRaj, Madhwa H.G.Basile, TeodoraJůzl, MiroslavRajabzadeh, Amin RezaBastarrachea, Luis J.K. Karabagias, IoannisRajkovic, AndrejaBatista, Ana PaulaKahlon, Talwinder S.Raju, RiteshBattaglia, EricKairytė, AgnėRakicka-Pustulka, MagdalenaBatule, Bhagwan SahebraoKalambura, SanjaRamos, MarinaBautista-Gallego, JoaquinKaletunc, GonulRamos-Diaz, Jose MartinBavaro, Simona LuciaKalhotka, LiborRanadheera, Chaminda SenakaBeauchamp, JonathanKallas, ZeinRanadheera, SenakaBeccaria, MarcoKalogianni, DespinaRao, QinchunBednářová, MartinaKamenidou, IreneRapa, MattiaBeier, RossKamiloglu, SenemRapeanu, GabrielaBelghit, IkramKandylis, PanagiotisRasinger, Josef DanielBellido-Milla, DoloresKang, Hui-SeungRatusz, KatarzynaBellosta, PaolaKang, KyungsuRau, HsinBellumori, MariaKangas, MichaelRayner, MarilynBenedek, CsillaKantono, KevinRayson, Gary D.Bengoechea, CarlosKanzaki, HiroyukiReale, AnnaBenincasa, CinziaKarayannakidis, PanagiotisRebelo, JoãoBenítez Jiménez, José JesúsKarcz, DariuszRebollo-Hernanz, MiguelBenítez-Cabello, AntonioKarousou, EugeniaRegula, JulitaBentley-Hewitt, Kerry L.Kartheek, AnekellaReinoso-Carvalho, FelipeBeppu, FumiakiKartsonaki, ChristianaRembialkowska, EwaBeresford, TomKarus, AvoRemenyik, JuditBeretta, ClaudioKasapidou, EleniRemesar, XavierBergholz, TeresaKassama, LaminRen, Yuan (David)Berillis, PanagiotisKastner, HannaRenard, CatherineBerrue, FabriceKauffmann, Jean-MichelRendueles, ManuelBerski, WiktorKaurivi, Yolande BabyRescigno, AntonioBerthelsen, RagnaKawata, YukichikaResconi, VirginiaBerthold-Pluta, AnnaKay, Colin D.Restuccia, DonatellaBertoni, GiuseppeKebede, BiniamRevelou, Panagiota-KyriakiBetoret Valls, Maria EsterKeppler, JuliaRibeiro, Luís FilipeBevilacqua, AntonioKerekes, Erika BeátaRiccioni, GiuliaBezirtzoglou, EugeniaKetnawa, SunanthaRichetin, JulietteBhandari, Manohar PrasadKetto, IsayaRilcheva, Violeta S.Bhat, ZuhaibfayazKeyzers, RobertRincon, Angela M.Białek, MałgorzataKhajehei, ForoughRío Segade, SusanaBianchi, GiuliaKhallouki, FaridRipari, ValeryBierzuńska, PaulinaKhanal, SupreetRipoll, GuillermoBilbao, DanielKhatiwada, JanakRipolles-Avila, CarolinaBimbo, FrancescoKhaw, Kooi YepngRisius, AntjeBjörkroth, JohannaKhoobchandani, MenkaRistic, RenataBlackwell, BarbaraKicińska, AlicjaRiu-Aumatell, MontserratBlandino, AnaKieliszek, MarekRiuzzi, FrancescaBlank, RalfKilcawley, KieranRivadeneira, PaulaBlasi, FrancescaKim, ChyerRivero-Pérez, M. DoloresBogdański, PawełKim, HekapRizzello, Carlo GiuseppeBoggia, RaffaellaKim, Kwang-OkRizzo, MilenaBohrer, Benjamin M.Kim, Mina K.Roberts, JulieBolhuis, DieuwerkeKim, YeonsooRochfort, SimoneBondoc, IonelKim, Young-JunRodov, VictorBonfanti, AngeloKimura, HidetoRodrigues, SandraBonnländer, BerndKinchla, AmandaRodríguez Bernaldo De Quirós, AnaBoqué, RicardKing, Joan M.Rodríguez Villanueva, JavierBordons, AlbertKingsley, DavidRodríguez, JaimeBorges, AnabelaKirkendall, LawrenceRodriguez, Oscar AlbertoBoukid, FatmaKitinoja, LisaRodríguez, SorayaBoziaris, IoannisKlempt, MartinRodriguez-Couto, SusanaBožović, MijatKleter, Gijs A.Rodríguez-Flores, M ShantalBrahma, SandrayeeKlupczynska, AgnieszkaRodriguez-Pulido, Francisco J.Brandt, Markus J.Knaapila, AnttiRohn, SaschaBravi, LauraKneifel, WolfgangRomalde, Jesús L.Bray, JeffKo, Wen-ChingRomano, PatriziaBrighenti, VirginiaKobus, ZbigniewRondeau-Mouro, CorinneBrinson, RobertKoch, WojciechRos Berruezo, GasparBrisson, GuillaumeKoenig, LauraRosa, Ambra R. DiBroadhurst, C. LeighKoenigstorfer, JoergRosa, MarcoBrownlee, IainKöhler, KarstenRose, PeterBrownlee, Iain AKohyama, KaoruRosenthal, AndrewBrugiapaglia, AlbertoKohyama, NorikoRosicka-Kaczmarek, JustynaBrychcy, EwaKokoska, LadislavRossi, FilippoBryla, PawelKonopka, IwonaRouhiainen, AriBrys, JoannaKoppel, KadriRoullier-Gall, ChloéBuchli, JürgKopsahelis, NikolaosRoussis, Ioannis G.Buchtová, HanaKorzeniowska, MałgorzataRouster, PaulBück, AndreasKoutelidakis, Antonios E.Routray, WinnyBuckley, M.Kovačević Ganić, KarinRouvinen, JuhaBuczkowski, BartoszKowalczewski, PrzemysławRóżycki, MirosławBudryn, GrażynaKowalczewski, Przemysław ŁukaszRóżyło, RenataBui, Duc CuongKowalik, JaroslawRudzińska, MagdalenaBukowski, MichalKowalska, HannaRuggeri, FrancescoBuksa, KrzysztofKowalska, KatarzynaRuhnke, IsabelleBurak, M.FurkanKozačinski, LidijaRuiz-Aceituno, LauraBustamante, María ÁngelesKozłowska (Kozlowska), MariolaRuiz-Capillas, ClaudiaBustamante, SoniaKraak, VivicaRumbos, ChristosBuvé, CarolienKrahe, JamesRumpunen, KimmoCabanillas, BeatrizKrasniewska, KarolinaRuocco, GianpaoloCachero-Martínez, SilviaKrisch, JuditRusinek, RobertCádiz-Gurrea, María De La LuzKrischek, CarstenRusso, PasqualeCais-Sokolińska, DorotaKról, JolantaRutkowska, JaroslawaCalabria, DonatoKról-Kilińska, ŻanetaRyan, Michael P.Calasso, MariaKrstanović, VinkoRybczyńska-Tkaczyk, KamilaCalín-Sánchez, ÁngelKrupa-Kozak, UrszulaRyu, Gi-HyungCallanan, Michael J.Ku, SeockmoRzepecka-Stojko, AnnaCamara, Jose SousaKubiak, KatarzynaRzymski, PiotrCamilli, EmanuelaKubicova, LenkaSabia, MichaelCamiña García, MercedesKubo, IzumiSachadyn-Król, MonikaCamire, MaryKubow, StanSagnelli, DomenicoCamire, Mary EllenKudłak, BłażejSainz-García, ElisaCampbell, Fiona M.Kudo, YutaSajdakowska, MartaCampbell, LydiaKukula-Koch, WirginiaSakač, NikolaCampestre, CristinaKulig, DominikaSakai, NobuyukiCampiglia, PietroKuligowski, MaciejSalafranca Lázaro, Francisco J.Campisi, BarbaraKulis, MichaelSalaheen, SerajusCano-Ibáñez, NaomiKumar, AnandSaláková, AlenaCapillo, GioeleKumar, PankajSalanţă, Liana ClaudiaCapozzi, VittorioKunkel, DanielSalas, Joaquín J.Cappa, CarolaKuo (Marie), Meng-ISalas, José BlancoCarapella, LauraKuo, Chia-HungSaldo, JordiCarballo, JavierKupka, TeobaldSaliba, AnthonyCarbone, KatyaKus, PiotrSalim, Nora Salina MdCardinali, AlessandraKusznierewicz, BarbaraSalonen, Anna SofiaCardullo, NunzioKutasi, KingaSalucci, SaraCaridi, AndreaLa Barbera, FrancescoSalvo, AndreaCarocho, Márcio SoaresLaaksonen, OskarSamanidou, VictoriaCarragher, JohnLabus, KarolinaSammartino, Maria PiaCarrera, Ceferino A.Lachman, JaromirSampietro, MarcoCarrubba, AlessandraLachowicz, SabinaSanchez, GloriaCarta, GianfrancaLaddomada, BarbaraSanchez-Zapata, ElenaCarullo, GabrieleLaganà, AldoSancho, M. TeresaCaruso, CalogeroLaganà, PasqualinaSanmartin, ChiaraCaruso, GianlucaLaghi, LucaSantiago, MichaelCaruso, Marisa C.Laguarda-Miró, NicolásSantillo, AntonellaCasal, SusanaLai, YandongSantini, AntonelloCasassa, Luis FedericoLamacchia, CarmelaSantos, CarlaCastada, HardyLamari, FotiniSantos-Gallego, CarlosCastro-Aceituno, VeronicaLambrecht, MarliesSaraiva, CristinaCataldi, T.R.I.Lambrecht, Marlies A.Saravana, Periaswamy SivagnanamCatzeddu, PasqualeLandi, MarcoSarkar, MitaliCayot, NathalieLandry, MatthewSarnoski, PaulCécile, MorrisLangowski, Horst-ChristianSatake, HonooCendrowski, AndrzejLangton, MaudSatake, MasayukiCerdá Suárez, Luis ManuelLappa, IliadaSatani, NikunjCerdá-Olmedo, EnriqueLaratta, BrunaSato, KenjiCervenka, LiborLaScalia, GiadaSaucier, CedricChambaron-Ginhac, StéphanieLatorre-Moratalla, Mariluz LuzSaw, Constance L. L.Chambers IV, EdgarLauro, Maria RosariaSawicki, RafalChandra, ShaneelLavelli, VeraSberveglieri, VeronicaChandrasekaran, MurugesanLe Thanh-Blicharz, JoannaScampicchio, MatteoChang, Hui-TingLech, KrzystofScarafoni, AlessioChang, Jae BongLee, AliceScarano, AureliaChang, Sheng-HsiungLee, Byung-HooSchendel, RachelChang, Te-ShengLee, Hee JaeScherf, Katharina A.Chang, Yu-WeiLee, Hwa-JinSchernthaner-Reiter, Marie HeleneChartier, AgnesLee, I-TaSchillaci, DomenicoChatzidimitriou, EffimiaLee, JeongmiSchippa, SerenaChen, BernardLee, Jun-SooSchiraldi, ChiaraChen, Bin-HueiLee, Ki-TeakSchmitt Olabisi, LauraChen, HuaiqiongLee, Sang-ahSchmitt, VéroniqueChen, Miaw-LingLee, Young SeongSchneider, FelicitasChen, Xuqi (Ricky)Lei, WeiSchottroff, FelixChen, ZhaoLeja, KatarzynaSchouteten, JoachimCheng, Kuan-ChenLenzi, AnnaSchreurs, NicolaCheng, Luisa W.Lesner, AdamSchulp, Jan A.Cheng, Yuan-BinLewandowicz, GrażynaSeifan, MostafaChern, Ming-KaiLewis, MikeSenadeera, WijithaChetwynd, AndrewLi, Fu-shuangSengupta, BidishaCheung, ShilinLi, GuijieSeoane-Collazo, PatriciaChien, Shih-ChangLi, MiaomiaoSeppä, LailaChilibeck, PhilLi, Yao OliveSepulcre, FrancescChin, Koo BokLi, YonghuiSerio, AnnalisaChiou, Tai-YingLiao, Hung-JuSetyaningsih, WidiastutiChiu, Wan-ChunLiao, Shu-ChuanSeverini, CarlaCho, SungeunLibera, JustynaSevilimedu Veeravalli, S.Choi, Jane RuLiberto, EricaShakerley, NicoleChoi, Jong-ilLiedl, KlausSharma, KavitaChoi, Sung EunLiguori, GiorgiaSharma, KumariChoi, Young MinLim, Kar HoShellie, R. A.Chung, SeojinLim, Tae-GyuShen, ZhenyuChuyen, Hoang V.Lima, Rita De Cássia LemosShete, VarshaCiaburro, GiuseppeLin, Chung-Tung J.Shiau, Chyuan-yuanCianfaglione, KevinLin, Hsu YangShimshoni, JakobCiardiello, Maria AntoniettaLin, Jin-YuarnShin, Hyun-JaeCicatiello, ClaraLin, Ming-KuemShrestha, SudhirCicero, ArrigoLin, ZhiweiSiano, FrancescoCichowska, JoannaLin, ZhuangshengŠic Žlabur, JanaCieślińska, AnnaLiou, Bo-KangSicari, VincenzoCione, ErikaListrat, AnneSigurdson, Gregory T.Cirkovic-Velickovic, TanjaLiu, ChangqiSikirzhytski, VitaliCiulu, MarcoLiu, JingSikorska, EwaClanchy, FelixLiu, JinmingSilva, Célia C. G.Claus, HaraldLiu, JunSilva, LuizClayton, Zachary S.Liu, RongmingSilva, Maria JoaoCobos, AngelLiu, Shing-HwaSilva, Severiano R.Coelho, AlfredoLiu, YanhongSilvério, Sara C.Coelho, ChristianLiu, YeyiSilvetti, TizianaCoelho, Denis A.Lizarazo, ClaraSim, KyraCoelhoso, IsabelLjubenkov, IvicaSimal, JesusCoisson, Jean DanielLlarena, Ann-KatrinŠimat, VidaColdea, TeodoraLo Scalzo, RobertoSimons, Courtney WayneColoretti, FabioLo, Chi-HungSinanoglou, VassiliaCompson, ZacchaeusŁobacz, AdrianaSingh, AnikaComstock, SarahLohan, SilkeSingh, Anubhav PratapConte, FrancescaLohumi, SantoshSingh, AshutoushConte, PaolaLomolino, GiovannaSingh, DeoContreras, María Del MarLončarić, AnteSingh, JaspreetCoombs, MelanieLongo, RoccoSipos, LászlóCorazzin, MircoLopes, GracilianaSipos, PéterCordero-Bueso, GustavoLopez Rodilla, Jesus M.Siren, HeliCorral Bobadilla, MarinaLopez, ArnoldoSirsat, SujataCorredig, MilenaLopez, FrancescoSitarek, PrzemysławCorreia, Ana CristinaLópez, MercedesSkenderidis, ProdromosCorreia, ManuelaLópez-Galán, BelindaSkendi, AdrianaCosentino, CarloŁopusiewicz, ŁukaszŚlusarczyk, SylwesterCossignani, LinaLores, MartaSly, TimothyCostantini, AntonellaLozano, JesusŚmigielski, Krzysztof B.Coulon-Leroy, CécileLu, Louise WeiweiSmith, Mark A.Cozzolino, DanielLu, ZhanhuiŚniegocki, TomaszCravero, Maria CarlaLu, Zhan-HuiSobczak, PawełCropotova, JannaLucarini, MassimoSobeh, MansourCrowe, JessicaLuccia, Aldo DiSobota, AldonaCrowley, Shane V.Lücke, Friedrich-KarlSocaci, SoniaCrupi, PasqualeLuckett, Curtis RobertSolano, FranciscoCruz, CarlosLuczaj, LukaszSoliven, ArianneCruz, Rui M. S.Luís, ÂngeloSolval, Kevin MisCruz-Chamorro, IvanLukic, IgorSommerer, NicolasCuadrado, CarmenLukinac Cacic, JasminaSommerhalter, MonikaCuomo, FrancescaLum, JuneSontag-Strohm, TuulaCuriel Gámiz, José AntonioLund, Marianne N.Sorrentino, E.Ćurko, NatkaŁyczko, JacekSoukoulis, ChristosCurto, BelénM. Pedrosa, MercedesSousa, ClaraCvrtila, ZeljkaMaak, Steffen MaakSovova, HelenaCzarniecka-Skubina, EwaMacaluso, MonicaSpence, CharlesD´Amico, StefanoMacedo, AntóniaSpizzirri, Umile GianfrancoD’Alessio, Patrizia A.Madhukar, BurraSpychaj, RadosławD’Antona, NicolaMaffei, GianlucaSrinivas, KeerthiD’Archivio, MassimoMaffia, MicheleSroka, ZbigniewD’huys, KarlienMafra, IsabelStaal, JensD’Incecco, PaoloMagalhães, Júlia M.C.S.Stadnik, JoannaDa Silva, A. MoreiraMah, Jae-HyungStagos, DimitriosDa Silveira Petruci, João FlávioMaicas, SergiStan, LauraDai, YuminMajchrzak, TomaszStanciu, SilviusDaima, Hemant KumarMakino, YoshioStanley, Jone A.Dalgaard, PawMakowska, AgnieszkaStanley, RogerDalmoro, AnnalisaMaleknia, Simin D.Starowicz, MałgorzataDalzini, ElenaMalfeito-Ferreira, ManuelStasiak-Różańska, LidiaDama, MuraliMalmberg, PerStathopoulos, CostasDanezis, GeorgeMalone, TreyStawny, MaciejDanijela Bursać, KovačevićMalvano, FrancescaStefanovic, BrankoDapkevicius, MariaMalysa-Pasko, MagdalenaStefanowicz-Hajduk, JustynaDavidovich-Pinhas, MayaMamone, GianfrancoStefanuto, Pierre-HuguesDe Bellis, PalmiraMancianti, FrancescaStella, SimoneDe Falco, BrunaMancini, SimoneStephens, JonathanDe Francesco, GiovanniMANDOLESI, SERENAStepien, AnnaDe La Fuente Blanco, AranchaMandrioli, RobertoStępień, SebastianDe Leonardis, AntonellaManes, JordiStessl, BeatrixDe Luca, GiuseppinaMangalathu, SujithSteup, MartinDe Lurdes Dapkevicius, MariaMangia, Nicoletta PasqualinaStirke, ArunasDe Masi, LuigiManiglia, BiancaStochelski, MateuszDe Pasquale, IlariaManjunath, ManuboluStorcksdieck Genannt Bonsmann, StefanDe Pilli, TeresaMannion, CynthiaStornaiuolo, MarianoDe Santis, DianaMannu, AlbertoStrassner, CarolaDe Smet, StefaanManthey, Frank A.Strobel, Scott A.De Vadder, FilipeMantzourani, IoannaStudzińska-Sroka, ElżbietaDe-Bandt, Jean-PascalMantzouridou, FaniStuper, KingaDegen, GiselaManuel, AleixandreStürup, StefanDegl’innocenti, DonatellaManuela, SannaSu, Nan-WeiDegola, FrancescaMaranghi, FrancescaSu, Wen-HaoDeiana, PierfrancescoMarchesini, GiorgioSubedi, KiranDel Caro, AlessandraMarchetti, NicolaSubramani Paranthaman, B.Del Coco, LauraMarciniak-Lukasiak, KatarzynaSudar, MartinaDelcour, Jan.Mariniello, LoredanaSugimoto, KeiichiroDelgado-Pando, GonzaloMaristella, GussoniSun, ChengwenDelogu, GiovannaMarizzi, ChristineSun, YvonneDeMarais, AlyceMarkaki, AnastasiaSung, I-HsinDeme, PragneyMarks, DavidSung, KidonDeng, LingzhuMarletta, LuisaSuomela, Jukka-PekkaDeo, PermalMarrone, RafaelleŠvecová, BlankaDepeint, FloreMarshall, JulieSvennevig, KatjaDerer, StefanieMarta, Ferreiro-GonzálezSwieca, MichałDerewiaka, DorotaMartin, RoseSzeleszczuk, ŁukaszDesai, Ajay S.Martin-Diana, Ana B.Szkolnicka, KatarzynaDi Grigoli, AntoninoMartinelli, TommasoSzpyrka, EwaDi Mauro, MariaMartines, EmilioSzulc, KarolinaDi Nunzio, MattiaMartínez Valdivieso, DamiánSzumny, AntoniDi Pasqua, Laura GiuseppinaMartinez-Monteagudo, Sergio I.Szweda, PiotrDi Pierro, ProsperoMartínez-Navarrete, NuriaSzwengiel, ArturDi Pizio, AntonellaMartinez-Rojas, EnriquetaTachibana, KatsuyasuDi Salle, AnnaMartínez-Turiño, SandraTaft, Diana H.Di Venere, DonatoMartinez-Villaluenga, CristinaTagliazucchi, DavideDíaz, MaríaMartinotti, SimonaTakahashi, KoretaroDifonzo, GrazianaMartins, Artur J.Takemori, HiroshiDijkhuizen, LubbertMartins-Lopes, PaulaTalati, ZenobiaDimitrellou, DimitraMarturano, ValentinaTalavera, MartinDobson, GaryMartuscelli, MariaTalon, RegineDomínguez Álvarez, EnriqueMarzec, AgataTan, Bee KDominguez, RubenMashima, RyuichiTan, ChenDonadelli, R.A.Masino, FrancescaTania Vergura, DonataDonno, DarioMastanjević, KristinaTao, YunwenDoyen, AlainMasullo, MariorosarioTaoka, YousukeDrzymała-Czyż, SławomiraMateo, JavierTappi, SilviaDu, ChenyuMaterska, MałgorzataTarragon Cros, ErnestoDuarte, Maria PaulaMatiadis, DimitriosTassou, ChrysoulaDubé, LauretteMatkowski, AdamTate, Rothwelle J.Dugmore, Thomas IainMatos, ManuelTawfike, AhmedDuh, Pin-DerMatsufuji, HiroshiTaylor, MatthewDurazzo, AlessandraMatwijczuk, ArkadiuszTaylor, SteveDurgo, KsenijaMau, GunnarTeixeira, NatérciaDwamena, AmosMaurer, PatricTemesi, AgostonDziki, DariuszMazzoni, EmanueleTemple, NormanDzunkova, MariaMcAuley, Catherine M.Tepper, Beverly J.Ehrmann, Matthias A.McMillin, Kenneth W.Terpou, AntoniaEiroa, José LuisMcNeil, DavidTeschke, RolfEl-Esawi, MohamedMcRae, Jacqui M.Tessier, Frederic JElias, MiguelMcSweeney, Matthew B.Theradiyil Sukumaran, AnurajEliopoulos, Panagiotis A.McWhorter, AndreaThomareis, ApostolosEller, FredMeacham, Susan L.Thomas, RaymondElls, TimothyMEDA, Naamwin-So-Bâwfu RomaricThomas, WayneEl-Seedi, HeshamMehli, LisbethThurner, ThomasElsinghorst, Paul W.Mekinić, Ivana GeneralićTidona, FlavioEngström, NiklasMelgar Castañeda, BrunoTiebe, CarloErcili-Cura, DilekMelini, FrancescaTinggi, UjangErmolina, IrinaMelini, ValentinaTirloni, EricaErsan, SevcanMello, Amilton DeTIXIER, Anne-SylvieEsatbeyolgu, TubaMenanteau-Ledouble, SimonTocmo, RestitutoEscott, CarlosMende, SusannTomczyk, MichalEspada-Bellido, EstrellaMendonca, AubreyTomczykowa, MonikaEsparza, M.Eugenia MartínMeneguzzo, FrancescoTorrico, Damir D.Espejo-Carpio, Francisco JavierMeneguzzo, FrancescoTørris, ChristineEsseili, Malak A.Menet, Marie ClaudeTosi, PaolaEsteves, EduardoMenga, ValeriaTovar, Clara Asuncion RodriguezEttayapuram Ramaprasad, Azhagiya SingamMenger-Ogle, AndrewTraczyk, IwonaEtzel, MarkMenzel, CarolinTrafiałek, JoannaEugenio, ApreaMenzel, Christopher M.Tran, TrongEvageliou, VasilikiMercader, JosepTrani, AntonioEvans, Franklin D.Merlino, Valentina MariaTrif, MonicaEvon, PhilippeMesnage, RobinTrifol, JonFaccia, MicheleMhd Jalil, Abbe MaleykiTripodi, GianlucaFadda, AngelaMiao, TianxinTríska, JanFailler, PierreMiceli, NataliziaTroise, Antonio DarioFalcão, Soraia I.Micevski, MilenaTrujillo, Antonio-JoséFanelli, Rosa MariaMichalska, AnnaTruzzi, CristinaFang, ZhongxiangMichel, FrederickTrzaskowska, MonikaFaour-Klingbeil, DimaMichel, MaximilianTsai, Shu-YaoFarag, KarimMiddendorf, DanaTsironi, TheofaniaFaraone, ImmacolataMiele, Nicoletta AntonellaTsochatzis, Emmanouil DFarber, MatthewMiglietta, Pier PaoloTsopelas, FotisFares, ClaraMikołajczak, BeataTsopmo, ApollinaireFarsa, OldřichMildner-Szkudlarz, SylwiaTsuji, MasaharuFaure, ChrystelMilella, LuigiTudela, JoseFeke, Donald L.Mills, KerryTufarelli, VincenzoFeldmeyer, PierceMincione, AntonioTufariello, MariaFeng, SiyuMinjares-Fuentes, Jose RafaelTung, Tao-HsinFenton, HaeleeMirzaei, AliTunick, MichaelFeresin, Rafaela G.Mis, AntoniTurner, Paul J.Ferguson, LouiseMisciagna, GiovanniTuuri, GeorgianaFernandes Lemos Junior, Wilson JoséMishra, Geetesh K.Tyler, RobertFernández Barbero, GerardoMishra, Geetesh KumarTylkowski, BartoszFernández Espinar, TeresaMishyna, MaryiaUeta, IkuoFernández Uclés, DomingoMita, GiovanniUllah, AmanFernández, MarioMitkowski, PiotrUlusoy, BeyzaFernández-Muíño, Miguel AngelMitrus, MarcinUpadhyay, Rakesh K.Fernández-Ochoa, ÁlvaroMitulovic, GoranValdes García, ArantzazuFernández-Soto, PedroMiyazato, HironariValoppi, FabioFerreira, Ana M.Mlček, JiriVan Buiten, CharleneFerreira, JenniferMoate, Peter J.Van Buul, Vincent J.Ferreira, SusanaMoatsou, GolfoVan De Walle, DavyFerrer-Gallego, RaúlMohamed, Azza H.Van Der Kamp, Jan WillemFerrer-Mairal, AnaMollers, ChristianVan Der Meeren, PaulFeveile Young, JetteMołoń, MateuszVan Kerckhove, AnneleenFigiel, AdamMonika, Waksmundzka-HajnosVan Loo, EllenFilannino, PasqualeMontesano, DomenicoVan Pamel, ElsFink, Ryan C.Montserrat, Costa-FontVan Spil, Erwin (W.E.)Fiorati, AndreaMoon, BoKyungVanesa, Benítez GarcíaFitsiou, EleniMoore, Carla J.Ványolós, AttilaFlanjak, IvanaMoore, KarenVáradi, JuditFlint, SteveMora, LeticiaVargas-Murga, LilianaFlorowska, AnnaMorais, Tiago G.Varzakas, TheodorosFocsan, Alexandrina L.Morales-Ramos, Juan A.Vasconi, MauroFogacci, FedericaMorán, LaraVaz, Daniela C.Fonseca, SoniaMorandini, Piero AngeloVazquez Espinosa, MercedesFörst, PetraMoreau, RegisVázquez, José AntonioFouladkhah, AliyarMoreira, Ana Sofia PereiraVázquez, ManuelFox, Mary A.Moreno García, JaimeVázquez-Araújo, LauraFracassetti, DanielaMoreno, VidalVecchiato, DanielFrancis, LeighMorgan, SydneyVejux, AnneFranco Ruiz, DanielMorozova, KseniaVemuri, RavichandraFrank, Damian C.Moschopoulou, EkateriniVenturi, FrancescaFranzoi, MarcoMossine, Valeri V.Verardo, VitoFraqueza, MaríaMotoki, KosukeVerger, EricFrias, JuanasMourtzinos, IoannisVerma, AnilFu, XueyanMozuraityte, RevilijaVerni, MichelaFuguet, ElisabetMuhlack, RichardVersari, AndreaFujiwara, AyaMuhle-Goll, ClaudiaVeskoukis, Aristidis S.Fusco, VincenzinaMuhovski, YordanVezzu, DileepGabr, MoustafaMulloy, BarbaraViapiana, AgnieszkaGabriella, PasiniMuniz, JuanVidal-Carou, CarmenGagaoua, MohammedMuramoto, KojiViktorova, JitkaGai, FrancescoMuriana, PeterVillalba, MayteGalán, Miguel LaderoMurru, ElisabettaVillalon, MarinaGalanakis, CharisMurru, NicolettaVirto, MailoGalani, Joseph Hubert YamdeuMusco, NadiaVisioli, FrancescoGalanis, AlexisMusto, MauroVitetta, LuisGalante, EvaMyracle, AngelaVoca, SandraGalego, LudovinaMyszkowska-Ryciak, JoannaVoliani, ValerioGallego, MartaNagano, TakaoVolny, MichaelGalus, SabinaNakata, HaruhikoVon Wright, AtteGambetta, Joanna M.Nakonieczna, AnnaVoznesenskaya, AnnaGamian, AndrzejNalewajko-Sieliwoniuk, EdytaWagenberg, C. P. A. VanGan, RenyouNanjundaswamy, AnandaWalker, DianeGanesan, PalanivelNapierala, MartaWalsh, MarieGangnat, IsabelleNardi, TizianaWalyaro, DJGao, BeiNardini, MirellaWang, BinGarbowska, MonikaNardon, ChiaraWang, DongyiGarcia Ivars, JorgeNarduzzi, LucaWang, GuorongGarcia Martos, Jose MariaNarsimhan, GanesanWang, KunGarcia, CyrielleNass, NorbertWang, PengchengGarcía-Camacho, FranciscoNatalello, AntonioWang, Qian JaniceGarcía-Díez, JuanNath, ArijitWang, YifeiGarcia-Gimeno, Rosa MariaNatskoulis, PantelisWanten, GeertGarcía-Martínez, EvaNavarro, CasildaWarren, FredGarcía-Salas, PatriciaNaviglio, DanieleWatanabe, MasanoriGardeli, ChrysavgiNawirska-Olszańska, AgnieszkaWatanabe, NaoharuGarmyn, AndreaNazzaro, FilomenaWatrelot, Aude AnnieGathercole, JessicaNelson, Stuart O.Watrous, JeramieGatti, MonicaNemzer, Louis RWeber, CarolynGaukel, VolkerNeunert, GrazynaWechsler, DanielGavahian, MohsenNguyen, Quang D.Weiler, UlrikeGawlik-Dziki, UrszulaNguyen, Quoc CuongWendin, KarinGazza, LauraNicoletto, CarloWhite, AlisonGélinas, PierreNieddu, GiovanniWia̧cek, Agnieszka EwaGerasopoulos, DimitriosNielsen, AndrewWianowska, DorotaGere, AttilaNiero, GiovanniWiczkowski, WieslawGésan-Guiziou, GenevièveNieva-Echevarría, BárbaraWierzbicka, ElżbietaGetty, KellyNikiforidis, Constantinos V.Wilde, Peter J.Ghosh, AbhijitNilsson, R. HenrikWilkinson, JennyGiaccone, ValerioNimalaratne, ChamilaWilkowska, AgnieszkaGiacometti, JasminkaNitsos, Christos K.Winnicka, KatarzynaGiamperi, LauraNitzko, SinaWitczak, MariuszGiannakas, ArisNiu, DongyanWitrick, KatherineGiannakourou, Maria C.Niwano, YoshimiWłodarczyk, MaciejGiannou, VirginiaNochera, Carmen L.Wójciak, KarolinaGiarratana, FilippoNolden, Alissa A.Wojdyło, AnetaGierszewska, MagdalenaNoordergraaf, Inge-WillemWojnar, WeronikaGil-Pérez, IgnacioNowak, AdrianaWojnowski, WojciechGimeno, Ana Cristina SánchezNowak, AgnieszkaWójtowicz, AgnieszkaGiribaldi, MarziaNowak, AnnaWojtyczka, RobertGiuggioli, Nicole RobertaNowak, DorotaWold, Jens PetterGiurg, MirosławNunes, CeciliaWołosiak, RafałGłąbska, DominikaNúñez Carmona, EstefaníaWong, MarieGodoy, Bibiana JuanNunez, OscarWood, JeffGogo, Elisha OtienoNychas, George-John E.Woźniak, ŁukaszGolinelli, Marie-PierreO’Keefe, Sean F.Wu, Shu-JuGolomazou, EleniO’Sullivan, MauriceXiong, JiaGomes, SóniaObrador, ElenaXu, GegeGómez, InmaculadaOchmian, IreneuszXu, ZhiminGonçalves, Elsa MargaridaOdeyemi, Olumide A.Yamaki, KohjiGonçalves, Fernando J.Odriozola-Serrano, IsabelYan, DayunGonçalves, SandraOhm, Jae-BomYang, ChengGondek, EwaOlafsdottir, GudrunYang, ChunGonzález Ariceaga, Citlalli CelesteOlech, MartaYang, Kai MinGonzalez Viejo, ClaudiaOlejar, Kenneth J.Yang, XiaoGonzález-García, EstefaníaOlejnik, AnnaYang, Yu-ChiaoGóral, TomaszOlejnik, MalgorzataYano, HiroyukiGordon, Eliza L.Oli, PrakashYazar, GamzeGori, AntonellaOlivan, MamenYe, LiyunGoroncy, AlexanderOliveira, AndreiaYeh, Shu-LanGorrasi, Ilaria Silvia RossellaOliveira, José M.Yoo, Hyun JuGórska, SabinaOliveira, SaraYoo, MiyoungGouws, Caroline A.Olleta, Jose LuisYoo, Sang-HoGrabez, VladanaOlmo-García, LucíaYoshihara, TatsuyaGrabowski, Nils Th.Olsen, AnnemarieYoshii, HidefumiGragg, Sara E.Olukosi, OluyinkaYou, Hyun JuGranato, DanielOmidian, KosarYu, Cheng-JuGranica, SebastianOniszczuk, AnnaYu, Chi-YangGrassi, SilviaOniszczuk, TomaszYu, Hsu-ShengGrasso, SimonaOracz, JoannaZacharis, Constantinos K.Greco, ClaudioOrdoudi, StellaZaferanloo, BitaGrieco, FrancescoOrecchio, SantinoZagorec, MoniqueGroh, Isabel Anna MariaOsek, JacekZaima, NobuhiroGrönman, KaisaO’Shea, NorahZalacain, AmayaGrossi Bovi Karatay, GrazieleOvissipour, RezaZanardi, EmanuelaGrossmann, ManfredPaciulli, MariaZappa, DarioGrosso, GiuseppePadalkar, MugdhaZarkada, AnnaGrzyb, JoannaPadhi, EmilyZawada, KatarzynaGuan, ViviennePadilla-Zakour, OlgaZdolec, NevijoGuedes-Alonso, RaycoPadrão, JorgeZdybel, EwaGuerra, Nelson PérezPaeng, KijungZettel, ViktoriaGuil-Guerrero, José LuisPaiva, LiseteZgórka, GrażynaGuinee, Tim PPająk, PaulinaZhang, BoGuinee, Timothy P.Panagou, E. Z.Zhang, ErshuaiGulden, ElkePandey, RenuZhang, QianGullon, BetatrizPanozzo, AgnesseZhang, YixuanGumul, DorotaPapadaki, AikateriniZhang, YuGuo, Chih-HungPapageorgiou, MariaZhang, ZhenbinGuo, JingshuPapoutsis, KonstantinosZhang, ZhiyunGurikov, PavelPappas, ChristosZhao, JingGustafson, Christopher R.Paramythiotis, SpirosZhu, ChangfuGutiérrez, Ana RosaParavisini, LaurianneZhu, FanGuzek, DominikaParente, EugenioZiarno, MałgorzataGyawali, RabinParenti, OttaviaZieniuk, BartłomiejHa, Jae-WonPark, Hyun JinZiino, GraziellaHa, MinhPark, SungkwonZimmerman, NathanHaase, HajoPark, Young-SeoZiuzina, DanaHackl, ThomasParlapani, Foteini F.Zoumpopoulou, GeorgiaHakonen, AronParmar, AdityaZych, MariaHalagarda, MichałParpinello, GiuseppinaŻyżelewicz, DorotaHalász, KatalinParras-Rosa, Manuel


